# Experimental evaluation of a cost-effective tesla turbine for waste air energy recovery in transportation systems

**DOI:** 10.1038/s41598-026-48846-z

**Published:** 2026-05-14

**Authors:** Mohamed B. Farghaly, Bandar Awadh Almohammadi, Abdullah M.A. Alsharif, Mohamed A. Mosbah, Eslam Hussein, Hamdy Abo El Daheb

**Affiliations:** 1 Mechanical Engineering Department, Faculty of Engineering, Fayoum University, El-Fayoum, 63514 Egypt; 2 Department of Mechanical Engineering, College of Engineering in Yanbu, Taibah University, Yanbu Al-Bahr, Saudi Arabia; 3 Electric Power Technology Department, Faculty of Technology and Education, Sohag University, Sohag, 82524 Egypt; 4 Mechanical Engineering Department, Faculty of Engineering at Shoubra, Benha University, Cairo, 11629 Egypt; 5 Automotive Technology Department, Faculty of Technology and Education, Sohag University, Sohag, 82524 Egypt

**Keywords:** Tesla Turbine, Waste energy recovery, Compressed air energy, Air brake systems, Energy harvesting, Energy science and technology, Engineering

## Abstract

Waste energy recovery from mechanical systems represents a promising approach for improving energy utilization in transportation applications. In air brake systems used in heavy-duty vehicles and railway trains, a considerable amount of compressed air is released without being utilized for useful energy conversion. This study presents the design, fabrication, and experimental evaluation of a cost-effective Tesla turbine intended for recovering compressed air energy from such systems. A bladeless Tesla turbine prototype was manufactured using CNC machining and consisted of ten coaxially arranged discs. Two disc materials, aluminum and steel, were investigated in order to examine the influence of material type on turbine performance characteristics. The turbine was integrated into a compressed air system and experimentally tested under inlet pressures ranging from 2 to 10 bar under both no-load and electrical load conditions. The rotational speed of the turbine and the generated electrical parameters, including voltage, current, and electrical power output, were measured and analyzed. The experimental results indicate that increasing inlet pressure leads to a noticeable increase in turbine rotational speed and electrical power output for both disc materials. However, the turbine equipped with steel discs consistently produced higher rotational speeds and greater electrical power output compared with the aluminum configuration under the tested operating conditions. In addition, at lower inlet pressures, the turbine with aluminum discs was unable to generate measurable electrical output, whereas the turbine with steel discs maintained stable operation. Overall, the findings demonstrate the feasibility of using a low-cost Tesla turbine to recover compressed air energy that would otherwise be wasted in air brake systems. The proposed approach may contribute to improved energy utilization in transportation systems where low-pressure compressed air sources are available.

**Supplementary Information:**

The online version contains supplementary material available at 10.1038/s41598-026-48846-z.

## Introduction

In the world of mechanical and energy engineering, compressed air is one of the most widely used media for energy transfer and conversion, commonly referred to as pneumatics. This science relies on the compressible and expandable properties of air to generate the required motion and force, making it an essential component in many industrial applications, such as pneumatic control systems, robotics, production lines, railways, and even alternative power generation processes. The combination of mechanical systems to capture waste energy is one of the most important innovations that countries worldwide strive to take advantage of air brake systems are fitted to both trucks and railroads. Trucks and trains operate with an air brake system that relies on a combination of components, including a compressor, tank, cylinder assembly, and wheel brakes. When the brake tank is full, it releases excess pressure to prevent the tank from exploding. Design and implementation of an innovative secondary circuit within the train air system, to exploit the untapped mechanical energy resulting from the operation of the train’s mechanical compressor. Normally, the compressor charges the air tank used in the brake system, and when the pressure reaches the required level, it stops pumping air into the main circuit, but it does not stop rotating, because it is mechanical and feeds directly from the engine or transmission system of the train, which means that its kinetic energy continues to work without useful load. Directing the compressed air generated by the compressor to a secondary circuit containing a Tesla turbine during periods when the charging process of the primary circuit stops. The Tesla turbine is a bladeless type that designs and operates on the principle of boundary layer adhesion. Its evident advantages over conventional turbines, such as ease of design, operational simplicity, reliability, and compatibility with various fluid types. The turbine converts air energy into mechanical energy, which is then used to power an electric generator to produce electrical energy that can be used inside the train in secondary applications such as lighting, charging batteries, or storing them as a reserve. Among the promising technologies that rely on compressed air in power generation, the Tesla turbine (TT) stands out as an innovative and effective solution. Designed by Nikola Tesla during the early decades of the 20th century, this turbine features a unique working method based on viscosity and friction rather than the traditional blades of other turbines^1–7^. When compressed air is passed through closely spaced discs inside the turbine, the air energy is converted into mechanical energy with high efficiency, opening the way for its use in recovering wasted energy in industrial and railway systems. Integrating Tesla turbine technology with pneumatic systems represents a step towards developing sustainable solutions to exploit energy more efficiently. By utilizing compressed air wasted in industries or train systems, this untapped energy can be converted into an efficient source for generating electricity or operating various equipment. Several books and research explore the basic principles of pneumatics and explain the components of its systems, focusing on the Tesla turbine as one of the advanced applications that can contribute to enhancing the efficiency of using compressed air as a clean and renewable energy source. These books were discussing turbine design, operating mechanisms, possible applications, and ways to improve its performance to make the most of this promising technology. The Tesla turbine is promoted as a promising way to increase energy. For this specific use, TT is a regenerative system that reduces energy waste without sacrificing design requirements or system output quality, taking a step toward more environmentally friendly industries. A Tesla turbine simulation was done on a commercial program ANSYS Fluent package for the simulation of mass and heat transfer; sophisticated unstructured grid generation was utilized^8^. Tangential forces from turbulence and fluid viscosity, as well as the phenomena of fluid attachment to surface flowing past, provide the basis for the operation of the Tesla turbine. Descriptions are given of the flow unsteadiness and flow field structures that arise in the spaces between the discs of rotor. The power unit distribution on the discs is established, and the turbine power forecasts derived from numerical analysis by Tesla turbine simulation were done on the commercial program CFX 18 ANSYS. Various approaches are used to compare efficiency estimation^9^. Higher performance at lower rotational speeds is demonstrated by the Tesla turbine, that integrates computational and experimental fluid dynamics studies. A thorough thermodynamic model serves as the basis for the performance study of the waste heat recovery system under varied operating conditions. A waste heat recovery system’s overall thermal efficiency and power are enhanced over a range of low rotation-speed, according to the results. This can be further enhanced by choosing the right working fluid viscosity and nozzle count, which is done through an experimental study. Effectively increasing a car engine’s power output and thermal efficiency at a reduced cost and volume is desirable and significant^10^. Due to its many applications, the Tesla Turbine has become widely accepted as a renewable source of energy for its rapidly expanding industrial sectors. The design, build, and test of an efficient small-scale Tesla turbine for distant locations was investigated by experimentally studies. An alternating current or direct current generator may be utilized in conjunction with the Tesla turbine’s mechanical power to produce energy^11^. A comparison between the performance prediction and evaluation of the primary flow characteristics of a Tesla turbine operating with organic fluids was carried out using an in-house developed 2D computational code, complemented by simulations performed with commercial computational fluid dynamics (CFD) software. The fluid enter the turbine rotor as tangentially as possible, and the bladeless rotor was positioned inside a frame that contains one or more nozzles that allow the fluid enter the turbine system circumferentially^12^. The experiment is run with different loads and inlet pressures. Since it is essential to achieving high turbine power and efficiency, the rotor disc’s roughness is measured. The identical conditions as in the experiment are used for numerical analyses. Pressure and circumferential velocity distributions are overestimated by the analytical model^13^. In comparison with other types of prime movers, Tesla turbines generate electricity at a low torque and high rotation rate. This characteristic makes Tesla turbines unsuitable for dynamometers used to evaluate gasoline and electric-powered radio-controlled vehicles and kit cars on a small scale. Methods are required to facilitate the design and performance assessment of Tesla turbines. Carey recently developed an analytical modeling approach and dynamic dynamometry, a complementary experimental technique. The turbine is constructed using polylactic acid (PLA) 3D printing^14^. The compressed air powers the Tesla turbine. To investigate the effects of geometrical elements such as the diameter of the rotor and the distance between the housing and rotor, two prototype Tesla turbines with disc diameters of 15 and 11.25 cm were created. The rotor and casing are separated by 3 mm each. Specifically, the impact of pressure flow and disc spacing is examined for each turbine under a variety of actual operating settings. According to the numerical results, the maximum values of wall shear stress are found on the rotor’s disc periphery, and the isentropic power loss in the area between the housing and rotor can reach 36.17%^15^.

Low-pressure head Tesla microturbine design, construction, and testing. To scavenge energy from the fluid flows created in plant-like evaporative systems. Tesla turbines are very easy to construct, scale down very favorably, and exhibit great efficiency when operated with low-pressure flows, in contrast to conventional inertial turbines. To the best of our knowledge, this 1 cm^3^ rotor diameter Tesla turbine is the smallest one ever documented. It has a peak efficiency of 40% and achieved an unloaded peak power output of 45 mW at a flow rate of 12 cc/s with an efficiency of 17%^16^. One of the first expanders, the Tesla turbine, uses the wall shear stress mechanism to transmit torque. The concept, which has been demonstrated for the air expanders on a laboratory scale, has some appealing properties when used: it can be scaled to a practical size, with rotor diameters ranging from 0.1 to 0.3 m, capable of achieving considerable rotational speeds, and can accommodate moderate flow rates, similar to those found in machines operating within the 500 W to 5 kW range. Subsequently, the model based on compressible real fluid assumptions was applied to the organic working fluids to assess the performance of the Tesla turbine in this setting. In order to provide easy adaptation to the particular application, a primary goal is also to present a modular design for the machine^17^. Efficiency-based numerical optimization of the Tesla turbine was investigations. The procedure of optimization is applied to the following parameters: rotating velocity, pressure, nozzle angle, inter-disc spacing, and inlet nozzle height. To optimize the process, a response surface approach was used. As it is neutral to efficiency, the pressure ratio was not included in the first optimization stage. The second optimization stage made it possible to identify the most crucial parameters needed to maximize the turbine’s efficiency: the partial admission coefficient, ratio of tangential velocity, aspect ratio, and reaction degree. Efficiency increased nearly double compared to the nominal model, the efficiency increased from 9% to 17%, and this improvement was confirmed by experimentation. Using computational fluid dynamics software, the optimized turbine model’s performance parameters were determined^18^. A theoretical analysis is used to determine how the dimensionless machine characteristics affect efficiency and performance. Analytical methods are used to determine the bulk flow’s streamlines. After being calculated theoretically, the conditions of inflow for optimal efficiency and performance are compared to laminar CFD simulation model. The CFD is used to analyze various input conditions of operation and their effects on the flow behavior and shaft power to evaluate the error of the theoretical analysis in simplified form. A comparison is made between the distribution of axial velocity development at the entrance zone and the one derived from the assumption of theoretical inflow. The effects of revolution speed and Reynolds number on profiles of velocity are examined. Furthermore, a model of compressible flow is presented^19^. One feature that sets the Tesla turbine apart is its bladeless design, which facilitates straightforward manufacturing and operation. If an efficient design can be constructed, it presents a compelling alternative for power output in small and microscale systems. The 1-D model is helpful because it can accurately depict the characteristics of flow of Tesla turbine and facilitate parametric exploration during the preliminary design phase. The limit of nozzle’s expansion ratio, that is correlated with the working fluid’s characteristics and the geometry angle, is shown. The nozzle’s flow loss is assessed rather than relying on an empirical velocity coefficient^20^. Tesla turbine produces power through viscous entrainment and can be referred to as a friction, viscous, or bladeless turbine. Its appeal has grown in recent years as distributed power generation applications have increased. Despite the fact that this expander is not appropriate for large-scale power generation, its low cost and dependability make it a promising technology for low power ranges^21^. With a focus on both experimental validation and theoretical modeling across an energy applications range, a thorough synthesis of current developments in Tesla disc turbine technology was provided in several studies. The viscous-dominated, intricate dynamics of flow inside turbine rotor have been thoroughly studied using analytical models and CFD simulations, emphasizing the role of Coriolis, centrifugal, viscous forces, and inertial in the power extraction. Under ideal circumstances, isentropic efficiencies surpass 0.75, and performance is extremely sensitive to the disc shape, nozzle design, and spacing. By presenting new low-loss nozzles, torque measurement methods, and proving a 30% power boost using the nanofluids, experimental investigations support these conclusions^22^. To determine the ideal structure and parameters of Tesla turbine in operation, a 3-D simulation model is created and analyzed using the CFD method. The accuracy and robustness of simulation model is further validated by designing and building a prototype of a Tesla turbine and conducting experiments with compressed air to drive turbine. Isentropic efficiency and power are considered goal parameters, while important operational parameters such as inlet pressure, mass flow rate, and rotational speed are also considered. Subsequent assessments of the turbine structural parameters, such as nozzle width, nozzle height, disc spacing, disc diameter, disc thickness, and number of turbine discs, are conducted after a review of the Tesla turbine’s basic operational characteristics^23^. Several studies were carried out to evaluate the factors influencing Tesla turbine operation. The turbine was built using a 3-D printing and 10 closely spaced PLA round disks, each measuring 150 mm in disc diameter and 0.9 mm apart. The experimentally measured the operational rotational speed and torque data were used to compute the power output and turbine’s efficiency. According to our experimental results, the total output power of Tesla turbine was lower than that of conventional turbines. With an ideal disc spacing of about 0.9 mm and diameter of about 15 cm, the measured efficiencies varied from 32% to 70%. Future research into the development of the Tesla turbine should focus on improving the turbine’s efficiency by modifications to the disk geometry or the use of alternative working fluids^24^. An numerical and experimental investigation were conducted on a modified Tesla turbine that is actuated by a compressed air. To explore the implications of geometric parameters such as the disc diameter and the spacing between the housing and rotor, two prototypes of the Tesla turbine, with disc diameters of 11.25 cm and 15 cm, were fabricated. The experimental characterization, founded on thermodynamic analysis, facilitates the determination of optimal values of pressure flow and disc spacing, which are estimated to be 103.89 kPa and 0.9 mm, respectively. Based on numerical analyses, it is observed that the maximum wall shear stress values occur at the periphery of the rotor disc, and the isentropic power loss in the region between the housing and rotor may attain levels as high as 36.17%^25^. The data obtained from an experimental campaign utilizing R1233zd(E) were employed for the purpose of computational analysis. The turbine exhibited an efficiency level of 29% alongside an output power of 0.57 kW. A three-dimensional computational model was implemented to scrutinize the dynamics of fluid present within the stator, stator-rotor interstice, and rotor. The performance results obtained from the three-dimensional computational fluid dynamics analysis were found to be aligned with those produced by the analytical two-dimensional code, which operated under the assumption of uniform rotor admission. Subsequently, a comprehensive analysis was carried out for the selected test case, encompassing a series of simulations across varying thermodynamic conditions, which were then compared with the results from the two-dimensional analytical code and the experimental data^26^. A technology aimed at improving the systems efficiency utilizing inverse cycles has developed. They specifically focus on the characteristics of two-phase flow of a Tesla turbine, which they analyze through numerical methods. Two computational techniques are employed: one is a custom-built mathematical model, and the other involves computational fluid dynamics (CFD) using commercial software. The analysis is conducted on the two-phase (R404a) fluid across a wide range of the rotational speeds, plate roughness levels, and plate gap sizes. The findings from both methods are compared with existing experimental data from the literature, showing strong agreement. The finding indicate an average output power of 0.8 W and a torque of about 3.6 mN-m at a speed of rotational approximately 2000^27^. A novel application of a Tesla turbine (TT) to improve the energy efficiency of the refrigeration cycles was proposed. Thus, TT is defined as a regenerative system which minimizes the energy loss while maintaining output quality and design integrity, promoting sustainable industrial practices. A detailed 3D thermo-hydrodynamic analysis has been performed on the Newtonian compressible turbulent flow of a high-pressure methane throughout a Tesla turbine, evaluating diverse configurations and operational parameters. The findings indicate that practical design guidelines are aimed at aiding engineers in optimizing TTs for operating conditions, especially regarding output power, disc size, and rotational speed^28^. Research to develop a compact, efficient Tesla Turbine for a remote applications was conducted and validated through experimental studies. Consequently, a performance evaluation of the newly engineered Tesla Turbine for remote contexts has been executed. This investigation encompasses computational analyses and an experimental phase involving turbine fabrication. Solid Edge 2021 was utilized for three-dimensional modeling. The selected materials for modeling include ABS Plastic or Polylactic Acid (PLA). The proposed configuration features 51 disks, uniformly spaced at 3 mm intervals. This design resulted in a torque of about 19.85 N-m and an impressive efficiency of about 81%. It was observed that the stress at the rotor disk edges exceeds that at the center due to elevated centrifugal forces^29^. Some data from the 3D printed of a small 4-disk Tesla turbine with specified disk inner/outer diameters of about (11.54 and 24.85) ± 0.01 mm were used to contrast and verify various operation methods. Dynamic dynamometry forecasts a maximum output power of 0.122 ± 0.008 W, which is 36.9% higher than the Carey model’s prediction of 0.077 ± 0.015 W. More precise parameter measurements will bring these values closer together, as bounding assumptions were utilized. There are other descriptions of peculiarities in the operation of miniature Tesla turbines, such as the finding that the rotational velocity of the turbine deceleration over time is not linear^30^. An experimental and numerical study of the Tesla turbine was provided. The experiment is run with different loads and inlet pressures. Since it is essential to achieving high turbine power and efficiency, the rotor disc’s roughness is measured. The identical conditions as in the experiment are used for numerical analyses. The analytical model and the computational findings are contrasted. The experiment and CFD have a reasonably good agreement when performance attributes are compared^31^. The performance prediction and evaluation of a primary flow characteristics of a the Tesla turbine operating with an organic fluids were compared using an in-house 2-D code created in an EES environment and a simulation was run using commercial CFD software. To assess the associated performance parameters of turbine, three working fluids (R134a, R404a, and R245fa) were examined. The results from the in-house code and CFD simulations matched very well^32^.

**Fig. 1:**
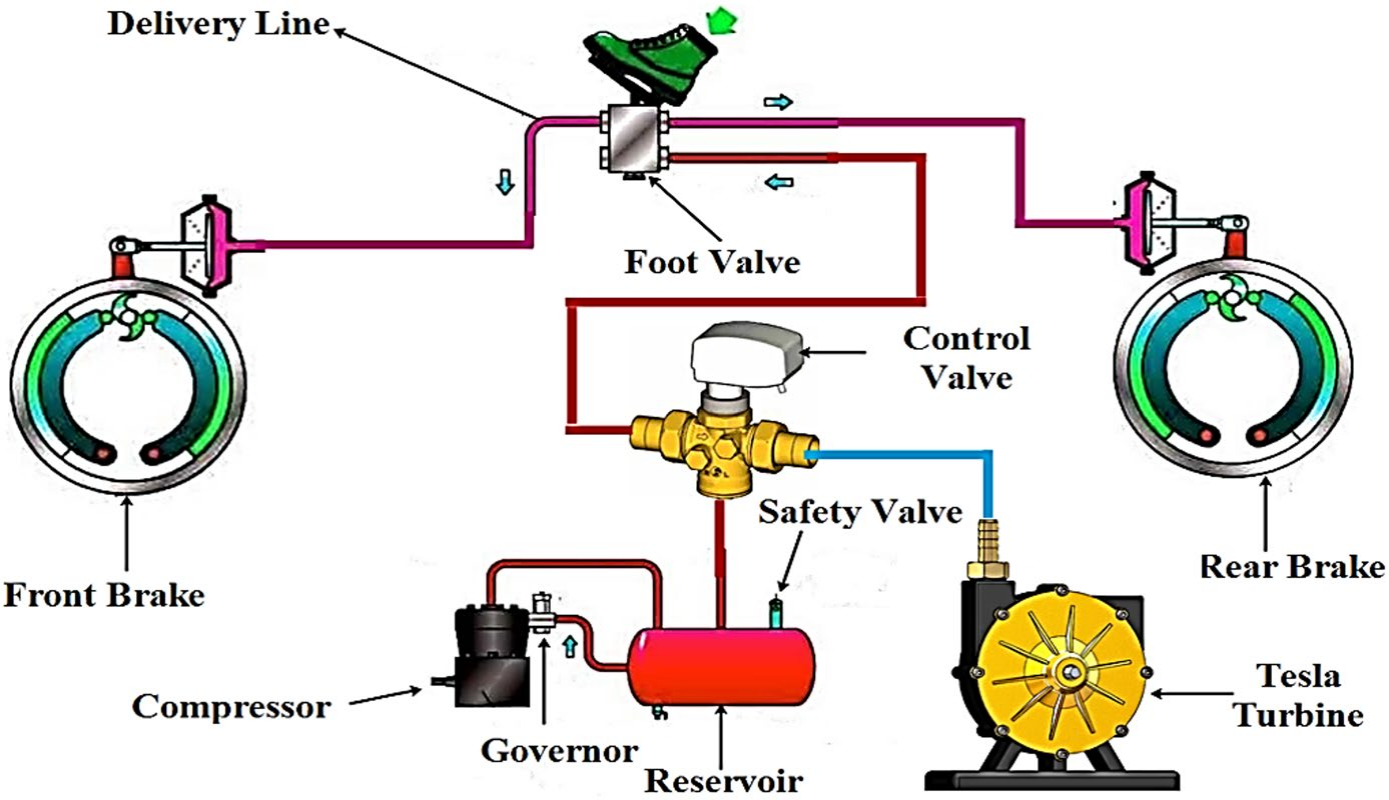
Air brake system with a Tesla Turbine installation.

In the current study, an experimental investigation was carried out on the Tesla turbine system to enhance understanding of its performance characteristics and practical applicability. This system helps increase the amount of energy resource utilization in trains by utilizing the continuous kinetic energy of the compressor during main circuit downtime, reducing loss and enhancing sustainability concepts, and rationalizing energy consumption in modern transportation. In this research, the Tesla turbine is designed to exploit the pressure wasted from the tank, and the air coming out of the compressor, if the tank is full, can also be exploited to generate electricity. When a Tesla turbine is installed in the pressure channel coming out of the tank or coming out of the compressor, this pressure will be used to generate electricity through the Tesla turbine. Tesla turbine was chosen due to its simplicity, as it relies in its operation on the effect of viscosity and friction between the air and the surfaces of the rotating discs, which makes it less sensitive to impurities and easier to maintain compared to traditional turbines with blades. The system consists of several integrated components, including a mechanical compressor, air flow control valves, heavy-duty hoses, a pressure gauge (manometer), a Tesla turbine discs, and an electric generator as illustrated in Fig. 1.

This study examines a cost-effective Tesla turbine prototype manufactured using CNC machining from two different materials, aiming to experimentally evaluate the impact of disc material and inlet pressure variations on its operational characterstics, emphasizing its suitability for operation in low-pressure environments. Especially in situations where conventional turbine systems are rendered impractical by cost or complexity. The outcomes of the current study are intended to guide future design improvements of Tesla turbines for applications including decentralized power generation and waste pressure recovery from the tank of rucks and trains that operate with an air brake system. During the experiment two types of blades (disks) with the same thickness of about 1 mm, and the same number of blades (10) were designed and fabricated, one made of aluminum and the other made of steel. Ten metal disks, each of about 150 mm in diameter and spaced of 0.9 mm apart, were concentrically arranged around turbine. With each blade type, the number of revolutions was measured for turbine shaft without and with loads. Also, an electrical generator was installed on the turbine shaft to measure current, voltage, power, and energy generated by the turbine. The practical experiment setup was done, and the measured performance parameters were evaluated on two phases. In the first phase, the Tesla turbine was assembled with its aluminum blades and the second was assembled from steel. The turbine inlet valve was connected to the compressor using heavy-duty hoses, and the air pressure was initially set at 2 bars and then the outside compressor pressure was then varied to 3, 4, 5, 6, 7, 8, 9, and 10 bar. At each pressure value, the turbine’s rotation speed was measured without load, as well as the turbine’s rotation speed with load (i.e., generator connection). The current generated by the generator was then measured using ammeter, the voltage generated was measured using an electrical ohmmeter, and the generated power capacity were also estimated and record simultaneously at the same time. In the second phase of the experiment, the aluminum blades were replaced with steel blades, and the turbine was assembled. The previous experiment was repeated to measure and evaluate the performance parameters. Figure 1 shows the overall air brake system with a Tesla turbine installation. The calibration of sensors were done by comparing the output results with another sensor has the same type and specifications, Additionally, multiple iterations of experimental tests were performed for each scenario to verify the accuracy of the obtained results. Despite the extensive body of research on Tesla turbines, most previous studies have primarily focused on theoretical modeling, numerical simulations, or performance optimization under controlled laboratory conditions. Limited attention has been given to the practical implementation of Tesla turbines for waste energy recovery in real engineering systems, particularly in transportation applications. Therefore, this study aims to address this gap by experimentally investigating a cost-effective Tesla turbine integrated within a compressed air brake system to recover wasted pressure energy. In addition, the study evaluates the influence of disc material (aluminum and steel) on turbine performance under low-pressure operating conditions, with a focus on electrical power generation. This approach provides new insights into the feasibility and practical applicability of Tesla turbines in decentralized energy recovery systems. Finally, it should be noted that the primary objective of this study is to evaluate the practical feasibility of a low-cost Tesla turbine for waste energy recovery applications, rather than to achieve full performance optimization or conduct a comprehensive efficiency analysis. The focus is placed on experimental validation under realistic operating conditions and assessing key performance trends.

## Mathematical model and operation principles

**Fig. 2:**
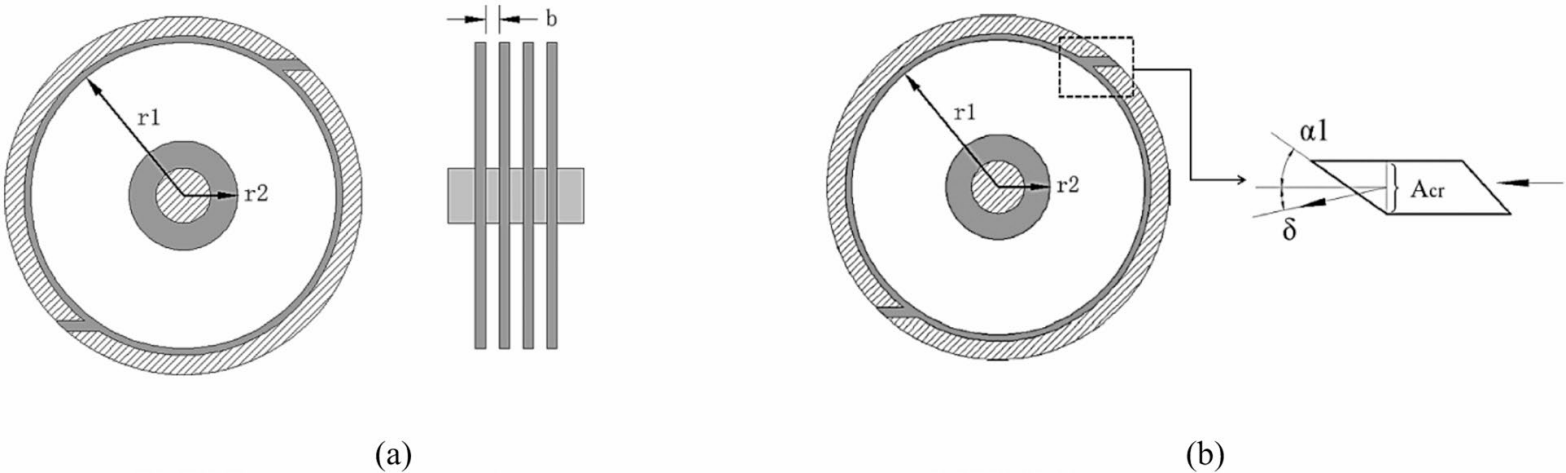
A Schematic diagram of a Tesla turbine, (**a**) Turbine rotor, and (**b**) Turbine nozzle^20^.

Figure 2 illustrates the schematic representation of Tesla turbine modeling considered in the current work. Initially, the working fluids are introduced into the turbine nozzles, wherein it undergoes expansion before subsequently flowing in a nearly tangential manner into the rotor blades (discs). The viscous effects arising within the boundary layers induce rotational motion of the discs. This throughflow in inter-disc spacing engenders a transfer of momentum between the discs and working fluid; consequently, power output and shaft torque are produced. The exhausted working fluid subsequently moves radially outward throughout the turbine outlet ports positioned adjacent to rotor shaft. The foundational model of the Tesla turbine has been elucidated to encapsulate the characteristics of flow in prior research^20^ that investigates multiple enhancements to the original turbine model. In analysis of nozzle, the limiting expansion ratio of oblique cut is duly considered as described in Eqs. (1–3). In rotor analysis, the governing equations pertinent to compressible flow are employed, and the friction factor between discs and working fluid is ascertained via Reynolds number as described in Eqs. (4–5). Furthermore, the pressure gradient in radial direction is taken into account, which is perceived as a driving force facilitating the movement of the working fluid from rotor inlet to outlet.

1 $$\:\dot{m}={A}_{cr}\times\:{\rho\:}_{cr}\times\:{a}_{cr}=\frac{\pi\:\times\:{d}^{2}}{4}\times\:\sqrt{{k\left(\frac{2}{\left(k+1\right)}\right)}^{\frac{k+1}{k-1}}}\times\:{p}_{^\circ\:}\times\:{\rho\:}_{^\circ\:}$$

2 $$\:\dot{m}={A}_{1}\times\:{\rho\:}_{1}\times\:{c}_{1}=\frac{\pi\:\times\:{d}^{2}}{4}\times\:\frac{\mathrm{sin}(\alpha\:+\delta\:)}{\mathrm{sin}\alpha\:1}\sqrt{\left(\frac{2k}{\left(k-1\right)}\right)\left[{\left(\frac{{p}_{1}}{{p}_{^\circ\:}}\right)}^{\frac{k}{2}})-{\left(\frac{{p}_{1}}{{p}_{^\circ\:}}\right)}^{\frac{k+1}{k}})\right]}$$

3 $$\:\mathrm{sin}{\alpha\:}_{1}\times\:\sqrt{{\left(\frac{2}{\left(k+1\right)}\right)}^{\frac{k+1}{k-1}}}\:=\mathrm{sin}\left({\alpha\:}_{1}+\delta\:\right)\times\:\left(\frac{2k}{\left(k-1\right)}\right)\left[{\left(\frac{{p}_{1}}{{p}_{^\circ\:}}\right)}^{\frac{k}{2}})-{\left(\frac{{p}_{1}}{{p}_{^\circ\:}}\right)}^{\frac{k+1}{k}})\right]$$

4 $$\:\frac{1}{r}\frac{{\partial\:}_{\left(rp{v}_{r}\right)}}{{\partial\:}_{r}}+\frac{1}{r}\frac{{\partial\:}_{\left(p{v}_{\theta\:}\right)}}{{\partial\:}_{\theta\:}}+\frac{1}{r}\frac{{\partial\:}_{\left(p{v}_{z}\right)}}{{\partial\:}_{z}}=0$$

5 $$\:{v}_{r}\frac{{\partial\:}_{{v}_{r}}}{{\partial\:}_{r}}+\frac{{v}_{\theta\:}}{r}\frac{{\partial\:}_{{v}_{r}}}{{\partial\:}_{\theta\:}}+{v}_{z}\frac{{\partial\:}_{{v}_{r}}}{{\partial\:}_{z}}=-\frac{1}{\rho\:}\:\:\left(\frac{{\partial\:}_{p}}{{\partial\:}_{r}}\right)+\mathrm{v}\left[\frac{1}{r}\frac{\partial\:}{{\partial\:}_{r}}\left(r\frac{{\partial\:}_{vr}}{{\partial\:}_{r}}\right)+\frac{1}{{r}^{2}}\frac{{{\partial\:}^{2}}_{vr}}{{\partial\:\theta\:}^{2}}+\frac{{{\partial\:}^{2}}_{vr}}{{\partial\:z}^{2}}-\frac{{v}_{r}}{{r}^{2}}-\:\frac{2}{{r}^{2}}\frac{{\partial\:}_{v\theta\:}}{{\partial\:}_{\theta\:}}\right]+{f}_{r}$$

Where; $\:\dot{m}$ represent the rate of mass flow in kg/s, p is the pressure in kPa, v is the velocity in m/s, r is the radius in mm, c is the sonic speed in m/s, A is the area in m^2^, k is the specific heat ratio, and f is the friction force.

## Problem statement

An innovative secondary circuit was devised and implemented within the train’s air management apparatus to utilize the mechanical energy that remains unutilized, which is generated by the train’s mechanical compressor. Generally, the compressor is tasked with replenishing the air reservoir employed in the braking system. Upon attaining the designated pressure threshold, the compressor ceases to inject air into the primary circuit. However, it continues to rotate due to its mechanical characteristics, receiving direct input from the locomotive’s engine or transmission apparatus. This operational characteristic suggests that its kinetic energy remains in motion without serving a constructive purpose. When the air pressure within the main reservoir rises to the upper limit of 10 bars, the internal mechanism of the switch compresses the electrical contact vane, thereby permitting electrical current to flow to the magnet. The magnet becomes magnetized, subsequently opening the air pathway to the unloading valves, which effectively depressurizes the charging cylinders and concludes the air compression cycle. In this state, the compressor continues to rotate without any discernible load. Following the consumption of some air, such as during braking or sling applications, when the pressure within the tank falls below 10 bars, the magnet relinquishes its magnetism, the lifting valves autonomously close, and the compressor initiates the air charging process. Based on this principle, the device proposed in this investigation channels the compressed air produced by the compressor into a secondary circuit that incorporates a Tesla turbine during periods when the primary circuit is dormant. This approach allows for the recovery of compressed air that would otherwise be lost without serving any functional purpose beyond the system. The turbine can convert the energy of the air into mechanical energy, which is subsequently harnessed to activate an electric generator, thereby producing electrical power that can be utilized within the train for auxiliary functions such as illumination, battery recharging, or energy storage. The selection of the Tesla turbine was influenced by its simplistic design and operational electrical performance. Its operation is based on the interactions of viscosity and friction between the air and the surfaces of the rotating discs, making it less susceptible to contaminants and facilitating maintenance, in contrast to traditional blade turbines.

## Tesla turbine components design and implementation

**Fig. 3:**
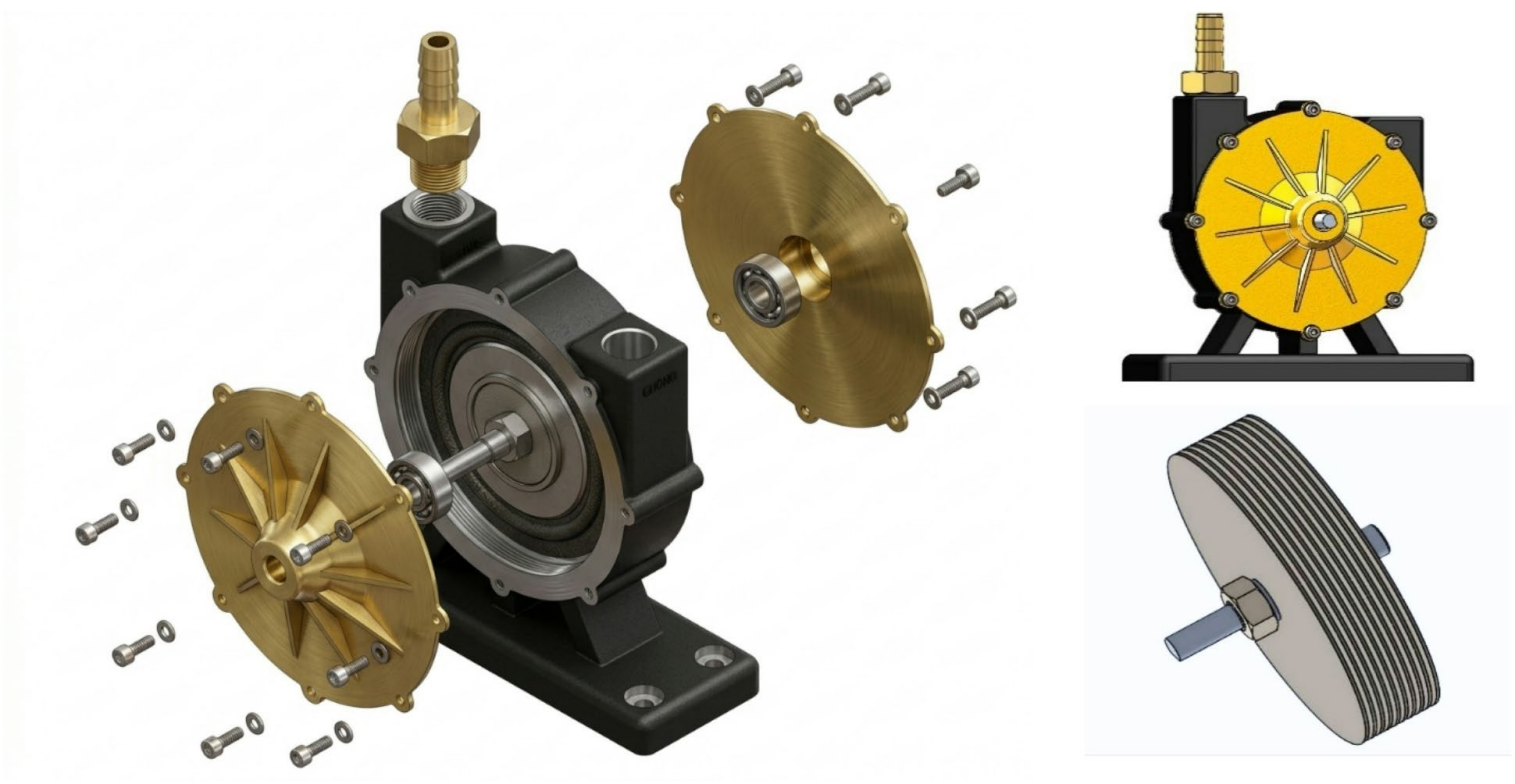
CAD design of the overall arrangement of Tesla turbine.

The core element of this system is the Tesla turbine, which captures pressure from the compressor and converts it into rotational motion, varying according to the applied pressure. Two types of 1 mm-thick blades one fabricated from steel and the other from aluminum were employed, while keeping the overall turbine dimensions constant. The blades were arranged at a fixed spacing of 20 mm. An experimental investigation was conducted to evaluate the influence of nine different inlet pressures on the turbine’s performance characteristics for both blade materials. The Tesla turbine wase design in the current work consists of a compact and modular assembly incorporating several key components. At the core, a rotor shaft supports a series of thin, closely spaced parallel discs, which form the bladeless turbine stage and are mounted concentrically to ensure uniform rotation. The shaft is anchored to the turbine housing through precision bearings, enabling smooth and stable operation. The housing itself is fabricated with a robust base plate for secure mounting and structural integrity. On either side of the disc stack, circular end plates are fixed, each fastened with multiple bolts to provide airtight sealing and structural rigidity. One of the end plates integrates an inlet nozzle, designed to direct compressed air tangentially onto the disc surfaces to exploit the boundary layer adhesion principle. Additionally, a series of outlet ports positioned at the disc center allow the working fluid to exit efficiently after transferring momentum. Many fastening screws, spacers, and sealing components were used to ensure accurate alignment, minimal leakage, and overall durability of the turbine system. Finally, the design was kept as simple as possible so that all parts could be easily manufactured and future improvements could be made to achieve optimized performance for energy recovery applications of a Tesla turbine system. The CAD design of the overall Tesla turbine assembly is illustrated in Fig. 3. This design highlights the simplicity, reliability, and adaptability of the Tesla turbine for energy recovery applications.

### Turbine shaft

**Fig. 4:**
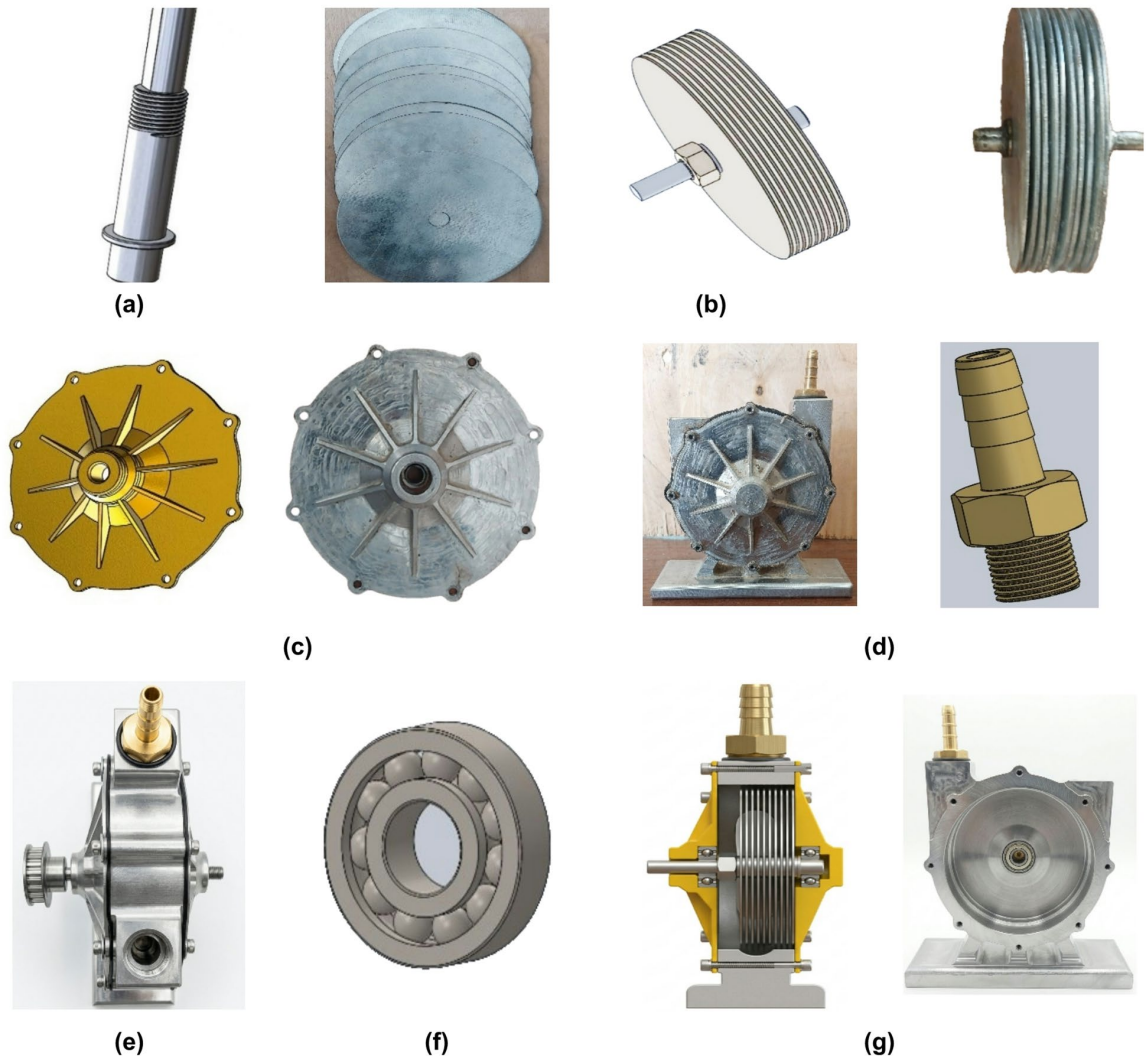
Design and CAD view of the manufacturing Tesla turbine components of all systems, (**a**) Turbine shaft, (**b**) Turbine blades (discs), (**c**) Turbine casing, (**d**) Inlet nozzle, (**e**) Exhaust port, (**f**) Bearing and fixing elements, (**g**) Turbine block.

A steel shaft (150 mm length, 10 mm diameter) supports the disc assembly and transmits rotational motion to the electrical generator. The material was selected to ensure sufficient strength and reliable torque transmission. The CAD design and the manufacutured real shaft is illustrated in Fig. 4(a).

### Turbine Blades (discs)

The rotor consists of ten parallel circular discs (150 mm diameter, 1 mm thickness) with an inter-disc spacing of 0.9 mm, constitute the primary energy-capturing element of the Tesla turbine. Aluminum and steel discs were tested to evaluate the effect of material properties on turbine performance. The discs convert the kinetic energy of the compressed air into rotational motion through boundary layer interaction. The CAD design and the manufacutured real turbine blades (discs) is illustrated in Fig. 4(b).

### Turbine casing

The turbine casing encloses the rotor assembly and ensures proper alignment and flow confinement. It also provides structural support and minimizes leakage and external disturbances. It features an outer diameter of 155 mm, an inner diameter of 10 mm, and a wall thickness of 3 mm. The material selection generally includes aluminum for lightweight applications or steel for enhanced strength and durability. The CAD design and the manufacutured real turbine casing is illustrated in Fig. 4(c).

### Inlet nozzle

The inlet nozzle is a precision-engineered component designed to regulate and direct the flow of compressed air into the Tesla turbine discs. A tangential inlet nozzle (20 mm diameter) directs compressed air into the turbine, generating a spiral flow that drives the disc rotation. Stainless steel are commonly selected materials due to their resistance to corrosion, high-pressure endurance, and durability under repeated loading cycles. The CAD design and the manufacutured real inlet nozzle is illustrated in Fig. 4(d).

### Exhaust port

The exhaust port (25 mm diameter) allows the air to exit the turbine after energy transfer, preventing backpressure and ensuring continuous flow. It is typically manufactured using precision drilling to achieve dimensional accuracy and surface smoothness. The material selection for the exhaust port generally matches that of the casing, commonly aluminum or steel, providing structural robustness, durability, and resistance to wear from high-velocity airflow. The CAD design and the manufacutured real exhaust port is illustrated in Fig. 4(e).

### Bearings and fixing elements

Bearings are used to support the shaft and reduce friction, ensuring stable and efficient rotation. It consists of an annular structure with an outer diameter of 90 mm and an inner bore diameter of 20 mm, dimensioned to accommodate the shaft while ensuring proper alignment and stability. The bearings are selected based on the standared roles to ensure dimensional accuracy, hardness, and fatigue resistance. Fixing elements maintain alignment and structural integrity of the system. The bearing and fixing elements is illustrated in Fig. 4(f).

### Turbine block

The turbine block serves as the main structural frame, housing all components and ensuring proper alignment and system stability during operation. It is characterized by a hollow cylindrical chamber centrally machined into a solid base, providing the necessary cavity for the disc assembly and shaft. The inner surface of the cavity is finished to minimize turbulence and reduce energy losses, while the outer surface is reinforced to withstand mechanical stresses and vibrations during operation. Aluminum alloys are often selected due to their lightweight, good machinability, and resistance to corrosion, while cast steel or stainless steel may be used where higher mechanical strength and durability are required. The CAD design and the manufacutured real turbine block is illustrated in Fig. 4(g).

## Experimental setup and operation procedures

### Experimental set-up

**Fig. 5:**
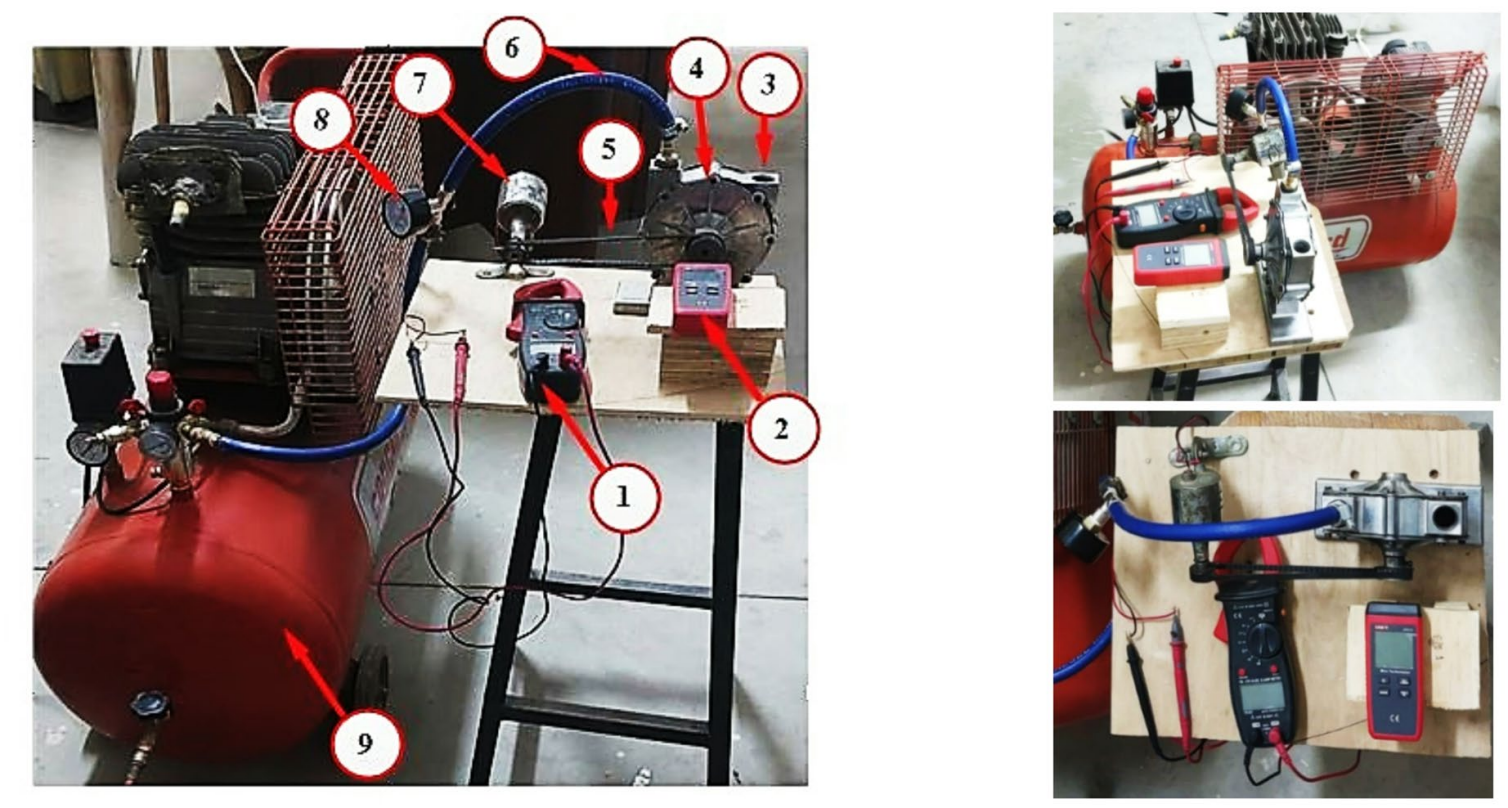
Layout of the experimental field setup for the tested unit and associated measurement instruments.

The system comprises several interconnected components, including a mechanical compressor, airflow regulation valves, robust hoses, a pressure gauge (manometer), turbine discs, and an electric generator. This configuration substantially enhances the electrical power output of energy utilization in vehicles by capturing the persistent kinetic energy of the compressor during periods of inactivity in the main circuit, thereby minimizing waste and promoting sustainability and energy conservation within modern transportation systems. In the current study, experimental tests of a Tesla turbine were designed and conducted on different days in May 2025, and the performance characteristics of turbine system were then evaluated. Figure 5 illustrates the configuration of the experimental field setup for the tested unit and Table 1 illustrates the measurement devices used during these tests. All experiment tests were manufactured, designed, and conducted in university workshops and laboratory, Egypt. Two types of blades (disks) with the same thickness of about 1 mm, and the same number of blades (10) were designed and fabricated, one made of aluminum and the other made of steel. Ten metal disks, each of about 150 mm in diameter and spaced of 0.9 mm apart, were concentrically arranged around the turbine. During each test for different blade types, measurements were recorded and taken of the number revolutions of turbine without and with loads. Also, an electrical generator was installed on the turbine shaft to measure current, voltage, power, and energy generated by the turbine. The studied Tesla turbine system was tested for different inlet compressor pressure of about 2, 3, 4, 5, 6, 7, 8, 9, and 10 bar. All parts of the tested turbine system were simultaneously operated to evaluate the overall enhancement of turbine system. The system’s output power was calculated to determine the optimal material type for same turbine configuration. The utilized sensors and devices calibration was carried out by comparing the output results with those obtained from other devices of the same type and specifications. Furthermore, to ensure the accuracy and reliability of the results, multiple experimental tests were performed for each scenario.

**Table 1:** Measuring instruments used during these tests.

No.	Measuring Instrument	No.	Measuring Instrument	No.	Measuring Instrument
**1**	Avometer	**4**	Belt	**7**	Inlet nozzle
**2**	Tachometer	**5**	Pulley	**8**	Exhaust port
**3**	Tesla turbine	**6**	Generator	**9**	Compressor

### Measurement Accuracy and Uncertainty Analysis

To ensure the reliability of the experimental results, each test was repeated three times under identical operating conditions. The reported values of rotational speed, voltage, current, and electrical power represent the average of these measurements. The variability of the measured data was quantified using the standard deviation, which was calculated for each parameter. Error bars representing ± standard deviation have been included to illustrate measurement consistency. The measurement instruments used in this study include a digital tachometer for rotational speed, a digital multimeter for voltage and current measurements, and a pressure gauge for inlet pressure monitoring. The accuracy of these instruments is within the typical ranges specified by the manufacturers (± 1–2% for electrical measurements and ± 1% for pressure measurements). These measures ensure that the experimental data are reproducible and that the observed differences between configurations are within a reliable measurement range.

### Operation procedures

**Fig. 6:**
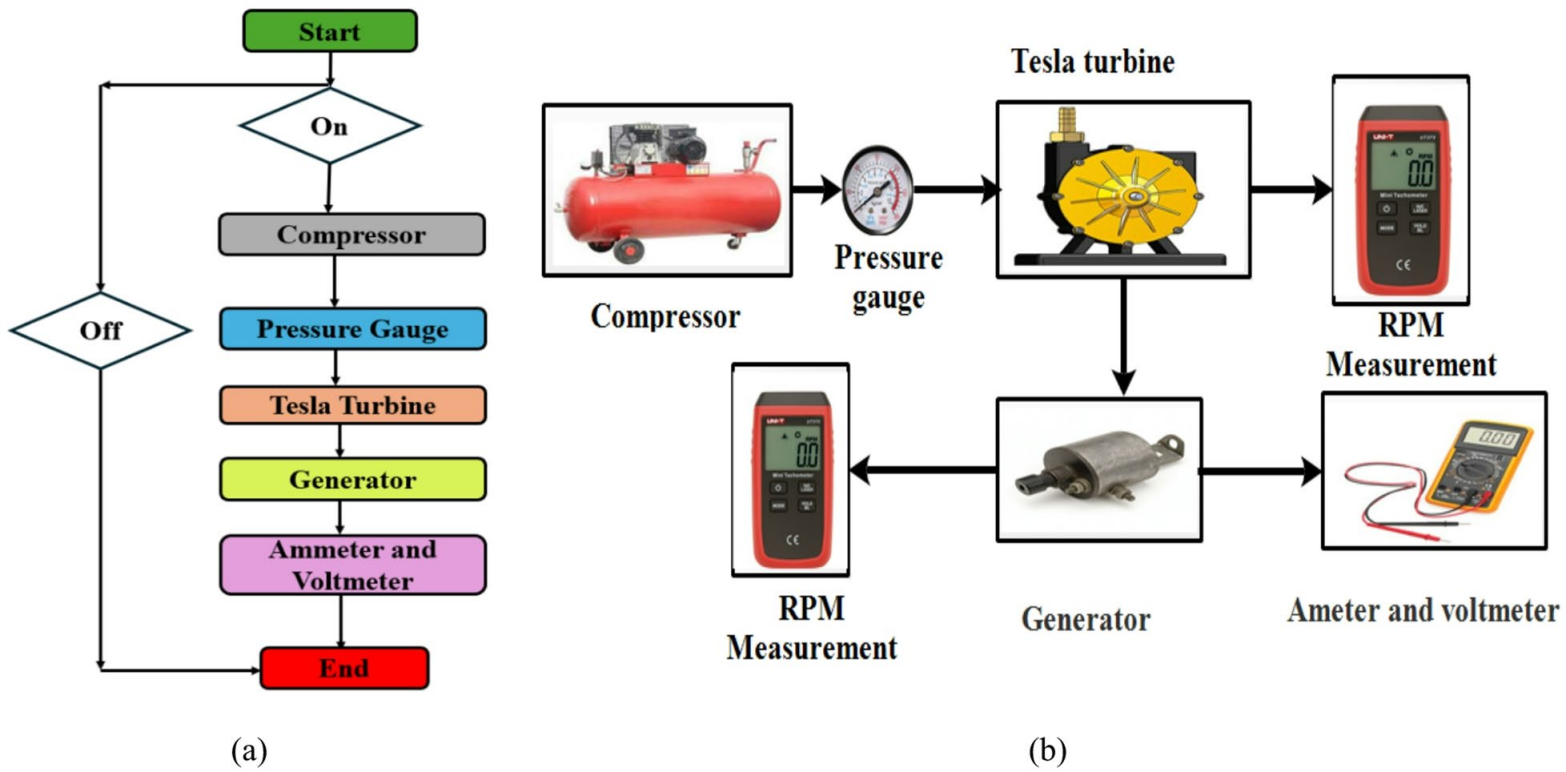
Flow chart of proceduers measurements during test days, (**a**) Flow chart, (**b**) Test proceduers.

The flow chart that illustrate the proceduers of measurements during test days for a Tesla turbine with an air brake mechanism within a tractor or truck was shown in Fig. 6(a). Upon the activation of the brakes to decelerate the tractor or truck, the compressor replenishes the brake reservoir due to the diminished pressure present within the reservoir. Conversely, when the pressure supply to the turbine is ceased, the turbine is stopped. Nevertheless, once the reservoir reaches its full capacity, the pressure adopts an alternative trajectory and progresses in the reverse direction. This pressure propels the turbine, which subsequently activates the generator. The generator transduces kinetic rotational energy into electrical energy, which can be measured by an ammeter and a voltmeter. Consequently, the otherwise expended air pressure may be wasted and transformed into electrical energy.

In the first phase, the Tesla turbine was assembled with its aluminum blades and the second was assembled from steel. The turbine inlet valve was connected to the compressor using heavy-duty hoses, and the air pressure was initially set at 2 bars and then the outside compressor pressure was then varied to 3, 4, 5, 7, 8, 9, and 10 bar. At each pressure value, the turbine’s rotation speed was measured without load, as well as the turbine’s rotation speed with load (i.e., generator connection). The current generated by the generator was then measured using an ammeter, the voltage generated was measured using an electrical ohmmeter, the power and generated capacity were also estimated and record simultaneously at the same time. In the second phase of the experiment, the aluminum blades were replaced with steel blades, and the turbine was assembled. The previous experiment was repeated to measure and evaluate the performance parameters. Figure 6(b) shows the overall air brake system with a Tesla turbine installation.

### Experimental model verification

**Fig. 7:**
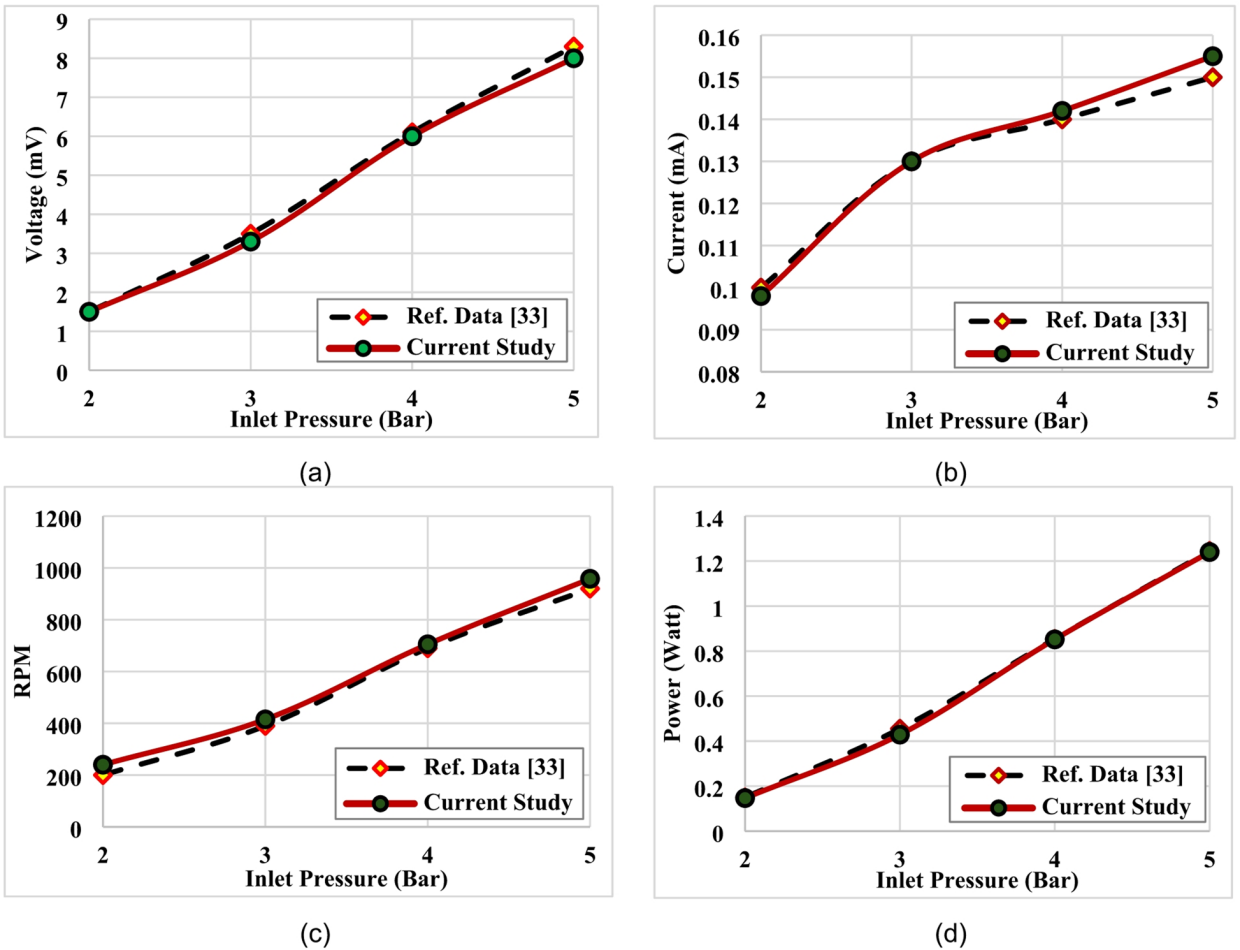
Experemental model validation, (**a**) Volte, (**b**) Current, (**c**) RPM, (**d**) Power^33^.

To validate the experemental model of tesla turbine system, the experemental results of the current study was compared with an experiment data as evaluated by Sumit Shinde and Hredeya Mishra^33^. The referance turbine is designed from a main shaft made of EN24, the casing material is aluminum, the turbine disc made of aluminum, and the front casing is also made of aluminum. The performance of the low-pressure recovery Tesla turbine, which is made of internal blades made of aluminum were evaluated, where low pressure from (2 to 5) bars can be effectively used to run the small generator. The generated power, volte, current were studied at different pressures, also, the effect of pressure changing on the turbine rotational speed were investigated. İn the current study, the parameters of experemental model of the case study been adjusted with the same operation conditions of inlet pressure of compared cases. The variation of power, volte, current, and rotational speed with different operation inlet pressure are illustrated in Fig. 7. The findings from experemental tests are compared with existing experimental data from the literature, showing acceptable agreement and good match of power, volte, current, and rotational speed. The error was originally calculated as the relative difference between the measured performance parameters in the present study and the corresponding value reported in reference^33^, using the following expression:

6 $$\:\text{Error (\%)}=\frac{\mid\:{X}_{\mathrm{exp}}-{X}_{\mathrm{ref}}\mid\:}{{X}_{\mathrm{ref}}}\times\:100$$

However, this comparison was based on a limited matching point rather than a full parametric validation, which may have led to an underestimation of the actual experimental uncertainty. A comparison with previously published results^33^ was conducted to assess the consistency of the experimental trends. Although some geometrical configurations are not identical, the case of turbine discs with (aluminum) material was selected and operating conditions were adjusted for low pressure from (2 to 5) bars to be identical with published results of reference. The observed relationship between inlet pressure and turbine performance characteristics shows qualitative agreement with the literature. Therefore, the validation is limited to confirming similar performance trends rather than providing a strict quantitative comparison. It should be noted that experimental uncertainties, including measurement accuracy and system variability, were considered in the analysis, and the validation results are interpreted within this context.

To estimated measurement uncertainty, a statistical analysis was conducted to ensure the reliability, repeatability, and consistency of the experimental results obtained from the Tesla turbine tests. All experiments were performed under controlled conditions and repeated three times for each operating point, including each inlet pressure level and disc material configuration (aluminum and steel). The reported values of rotational speed (RPM), voltage, current, and electrical power represent the arithmetic mean of the repeated measurements. The variability of the experimental data was quantified using the standard deviation (SD), calculated according to:

7 $$\:SD=\sqrt{\frac{1}{n-1}\sum\:_{i=1}^{n}({x}_{i}-\stackrel{\prime }{x}{)}^{2}}$$

**Fig. 8:**
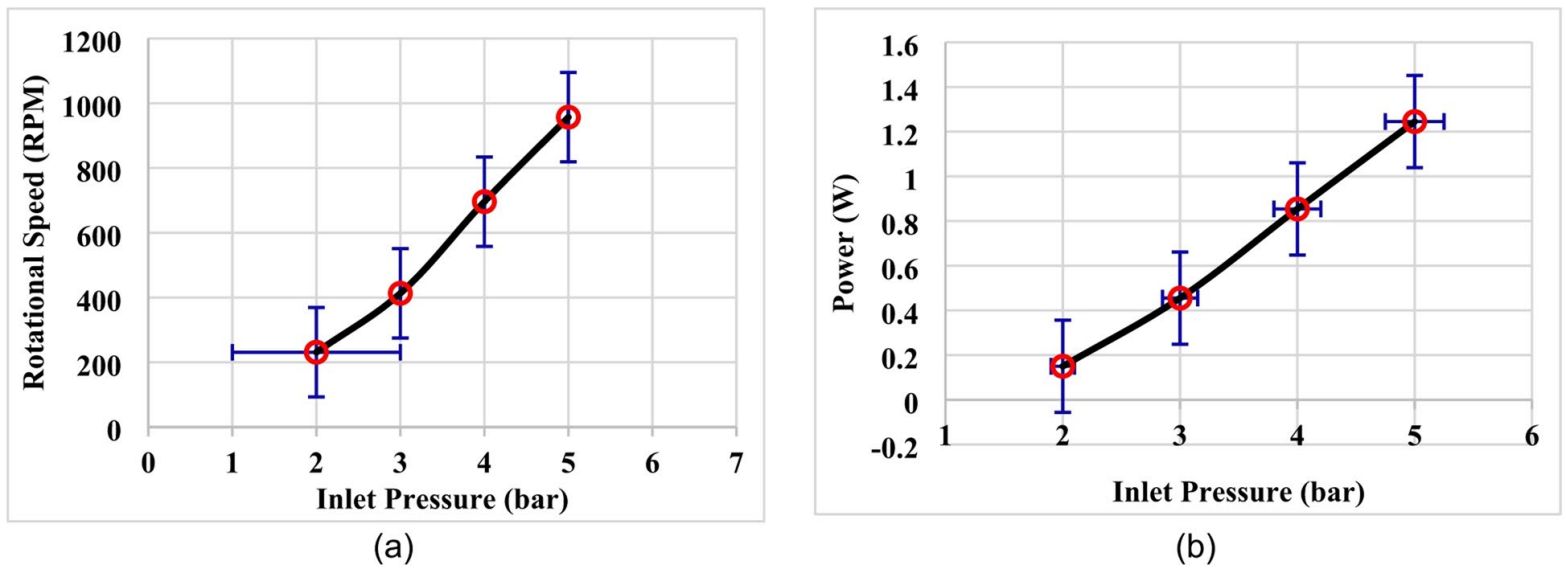
Variation of rotational speed (RPM) and electrical power with inlet pressure including Error bars represent ± standard deviation based on three repeated measurements, (**a**) Rotational speed (RPM), (**b**) Electrical power output.

Where $\:{x}_{i}$ represents each individual measurement, $\:\stackrel{\prime }{x}$ is the mean value, and $\:n$ is the number of repetitions. To visually represent the dispersion of the data, error bars corresponding to ± one standard deviation were included in the relevant plots, including rotational speed and electrical power as functions of inlet pressure as illustrated in Fig. 8; Table 2. Although the number of repetitions was limited, the results showed consistent trends across all tested operating conditions. The relatively small standard deviation values indicate good repeatability and consistency of the experimental measurements. Error bars representing ± standard deviation are included to illustrate measurement variability which calculated from three repeated measurements.

The accuracy of the measurement instruments was also considered in the uncertainty assessment. A digital tachometer was used for rotational speed measurements, while voltage and current were recorded using a digital multimeter, and inlet pressure was monitored using a calibrated pressure gauge. The measurement uncertainties associated with these instruments are within typical ranges specified by the manufacturers (approximately ± 1–2% for electrical measurements and ± 1% for pressure measurements). The combined effect of measurement uncertainty and experimental variability was found to be relatively small, supporting the reliability of the reported trends. However, it should be noted that a more comprehensive uncertainty propagation analysis and a larger number of repetitions could further improve the statistical robustness of the results. It is note that, the standard deviation values indicate good repeatability of the measurements, although slightly higher variability was observed at 3 bar, which may be attributed to transitional flow behavior within the turbine. To further assess the repeatability and reliability of the experimental measurements, the coefficient of variation (CV) was calculated for the rotational speed at each pressure level using Eq. (8). The obtained CV values ranged from 1.0% to 3.3% across the investigated pressure range (2–5 bar) as illustrated in Table 2. These values are well below the commonly accepted threshold of 5% for experimental studies, indicating good measurement consistency and low data dispersion. This confirms the reliability of the experimental procedure and supports the validity of the reported trends.

8 $$\:CV=\frac{SD}{Mean}\times\:100$$

**Table 2:** Measuring parameters during the validation tests.

Pressure (bar)	Mean RPM	Std Dev	Voltage (mV)	Current (mA)	Power (W)	CV (%)
2	≈ 240	≈ 7.9	1.5	0.1	0.15	3.3%
3	≈ 413	≈ 10.4	3.5	0.13	0.455	2.5%
4	≈ 705	≈ 8.5	6.1	0.14	0.854	1.2%
5	≈ 958	≈ 9.5	8.3	0.15	1.245	1.0%

## Results and discussion

To assess the operational effectiveness of a Tesla turbine characterized by dimensions of 150 mm in the outer diameter and 10 mm in the inner diameter, experiments were conducted using two sets of discs with identical geometric dimensions and thickness (1 mm), fabricated from aluminum and steel. The turbine was tested under nine inlet pressure conditions ranging from 2 to 10 bar. For each pressure level, the rotational speed of the turbine was measured under both no-load and load conditions. In addition, the electrical output parameters, including voltage, current, and electrical power, were recorded for a fixed operating period of 10 s.

**Fig. 9:**
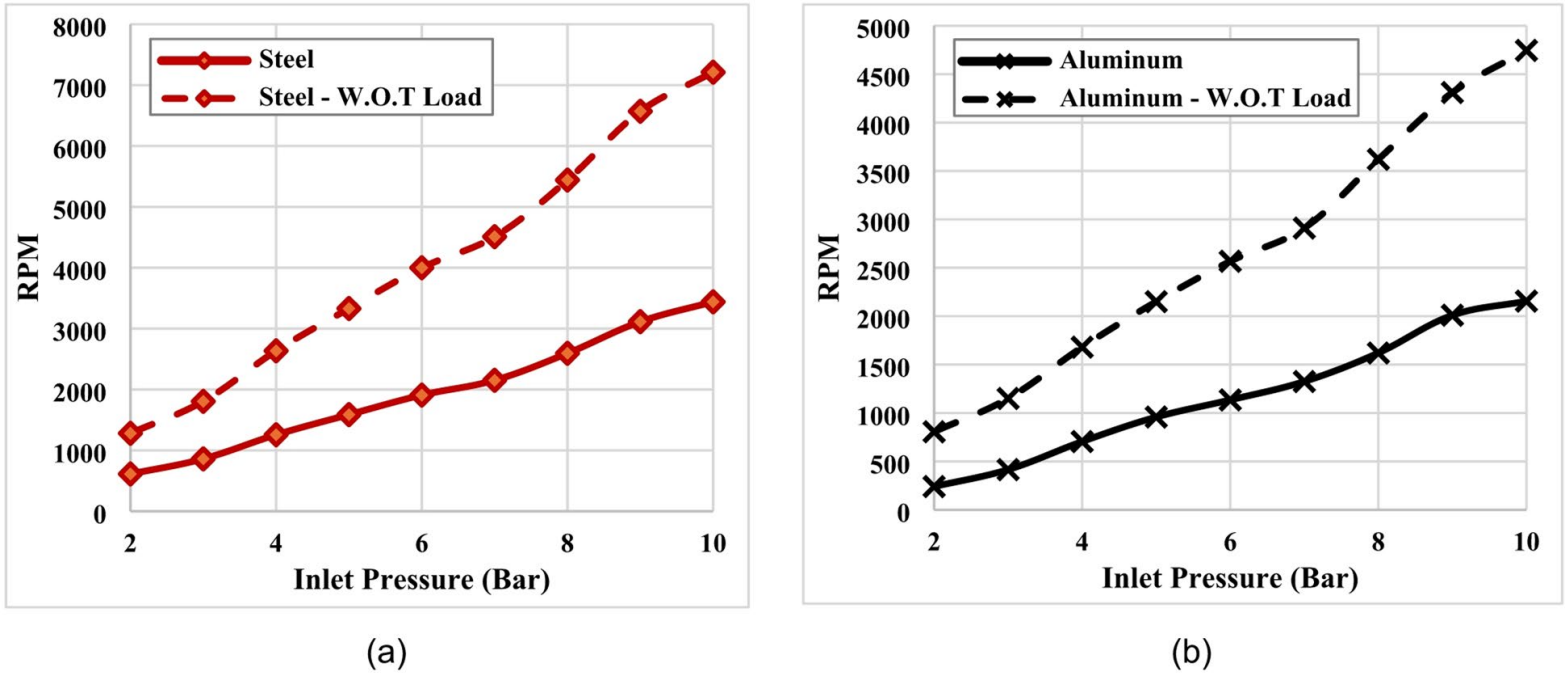
Variation of rotational speed (RPM) with inlet pressure under both loaded and unloaded operating conditions, (**a**) Steel blades (disc), (**b**) Aluminum blades (disc).

Figure 9(a) presents the relationship between pressure inlet and the rotational speed (RPM) of Tesla turbine equipped with 1 mm-thick steel blades, under both loaded and unloaded operating conditions. The results indicate a clear positive correlation between inlet pressure and turbine rotational speed, with higher pressures yielding greater rotational performance. Under no-load conditions, the turbine speed increased from approximately 1280 RPM at 2 bar to nearly 7212 RPM at 10 bar. Conversely, when the generator load was applied, the rotational speed decreased due to the opposing torque imposed by the electrical generator, ranging from about 612 RPM at 2 bar to approximately 3440 RPM at 10 bar. Despite this reduction, the turbine maintained a consistent upward trend with increasing pressure in both cases.

Figure 9(b) presents the corresponding results for the turbine equipped with aluminum discs. A similar relationship between inlet pressure and rotational speed (RPM) was observed with relative high reduction in rotational speed. Under no-load conditions, the turbine speed increased from approximately 860 RPM at 2 bar to nearly 4745 RPM at 10 bar. Under load conditions, the speed varied from about 240 RPM at 2 bar to approximately 2155 RPM at 10 bar. Despite this reduction, the turbine maintained a consistent upward trend with increasing pressure in both cases. These results demonstrate that the application of load reduces the turbine rotational speed in both configurations, while the general pressure–speed relationship remains similar. These findings confirm that while load application reduces overall rotational velocity, the Tesla turbine demonstrates stable and scalable performance characteristics, emphasizing its potential applicability in energy recovery systems operating under variable pressure conditions.

**Fig. 10:**
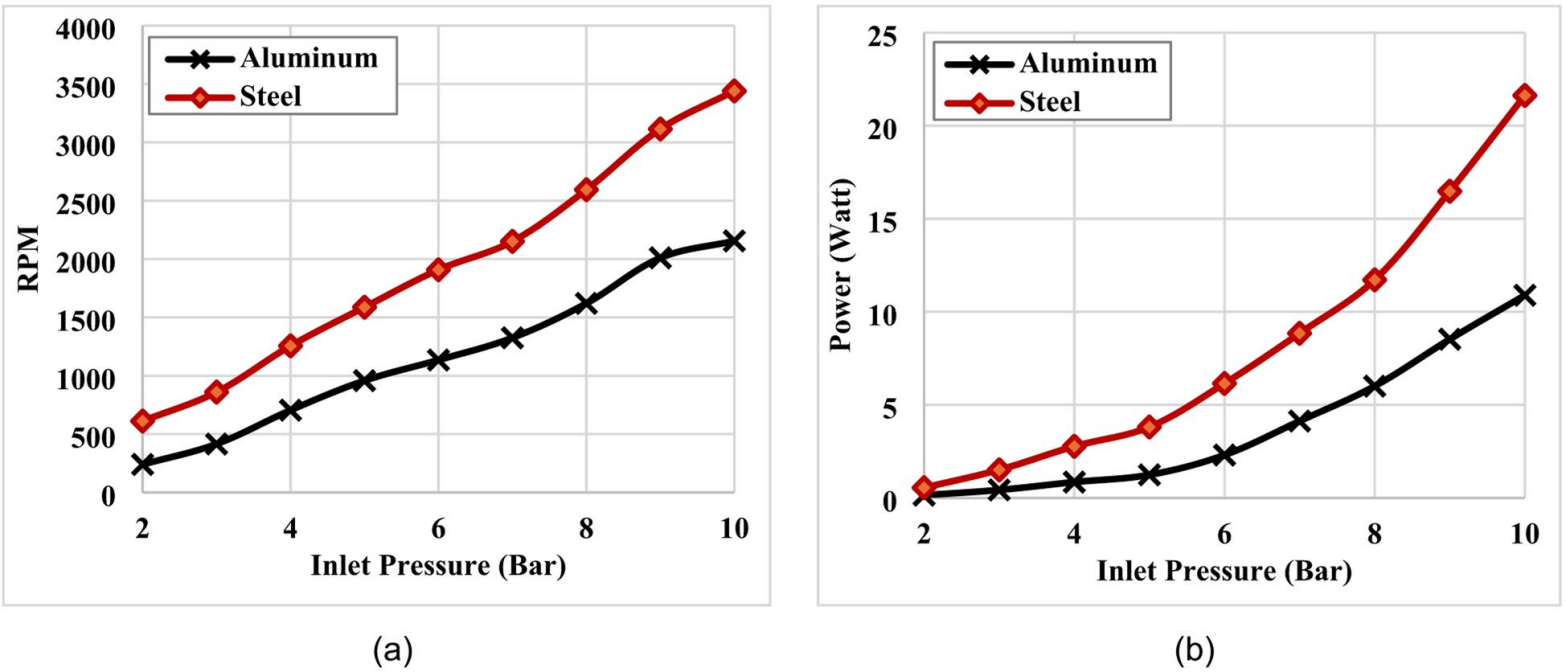
Variation of rotational speed (RPM) and output power with inlet pressure under loaded operating conditions, (**a**) RPM, (**b**) Aluminum blades (disc).

**Fig. 11:**
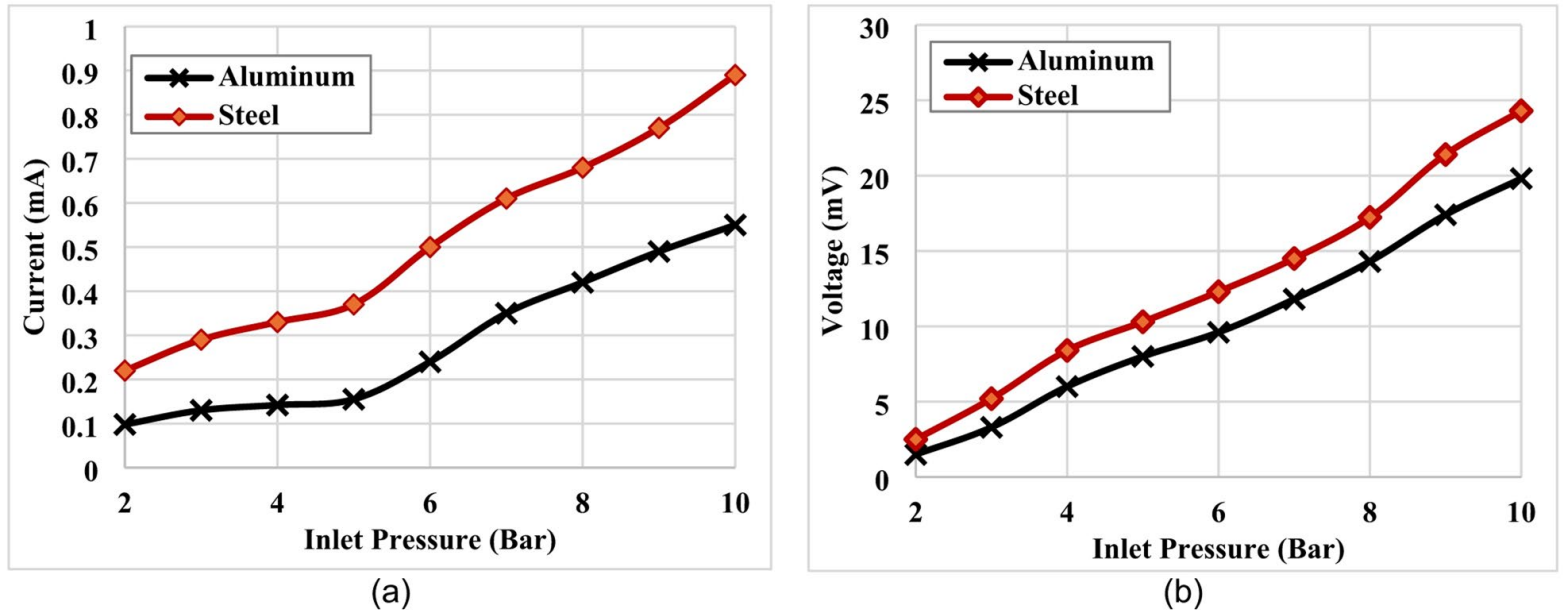
Variation of electric current and voltage with inlet pressure under loaded operating conditions, (**a**) Current, (**b**) Voltage.

To examine the influence of disc material properties on the turbine behavior, the performance trend of steel and aluminum configurations blades were compared with each other as illustrated in Fig. 10. and Fig. 11. The relationship between pressure inlet and rotational speed (RPM) of Tesla turbine for both configurations of steel and aluminum blades were compared with each other loaded operating conditions were illustrated in Fig. 10(a). While The relationship between inlet pressure and output power were illustrated in Fig. 10(b). Generally, a steel with its higher density and mechanical strength compared to aluminum, enables more effective momentum transfer from the compressed air to the rotating discs. This results in greater inertia and reduced energy losses, particularly under higher pressure conditions, which explains the higher RPM values recorded with steel. Moreover, in the case of aluminum blades, operation under low pressures (around 2 bars) fails to initiate rotation. However, as the pressure rises, the blades gradually begin to rotate, reaching a maximum speed of approximately 2150 rpm at 10 bars. In contrast, steel blades demonstrate the ability to rotate even at lower pressures, with their rotational speed increasing consistently as pressure rises, reaching a maximum speed of approximately 3440 rpm at 10 bars with percentage increasing of about 60%.

The variation of electrical power output with inlet pressure for both materials within the pressure range of 2 to 10 bar is illustrated in Fig. 10(b). In both cases, the electrical power increased progressively with increasing inlet pressure, demonstrating a clear positive correlation. However, the rate of increase is significantly higher for steel compared to aluminum. At relatively low inlet pressures (2–4 bar), the generated power remained small for both materials. As the pressure increased, the turbine equipped with steel discs exhibited a more pronounced increase in power output. At an inlet pressure of 10 bar, the electrical power generated by the steel configuration reached approximately 22 W, while the aluminum configuration produced around 11 W under the same conditions. This indicates that steel produces nearly twice the power of aluminum across the examined pressure range. The overall trend suggests that steel possesses better electrical performance and responsiveness to pressure changes than aluminum. This behavior may be attributed to the higher strength and structural rigidity of steel, which enhances its capability to convert pressure into mechanical or electrical power more effectively. This performance confirms higher electrical power output under the tested conditions of steel blades compared to aluminum blades, despite the latter’s lighter weight. Therefore, the experimental findings suggest that steel is the more suitable material for applications requiring higher electrical power output and durability, while aluminum may be advantageous in scenarios where weight reduction and ease of manufacturing are prioritized.

The measured electrical current and voltage as functions of inlet pressure are presented in Fig. 11. In both configurations, the electrical current increased with increasing inlet pressure as presented in Fig. 11(a). However, a clear difference is observed between the two materials. For the steel discs, the current rises more sharply compared to aluminum, starting from approximately 0.2 mA at 2 bars and reaching nearly 0.9 mA at 10 bars. This indicates a strong sensitivity of steel disc to inlet pressure variations. In contrast, aluminum disc exhibits a lower current response throughout the tested range, starting from around 0.1 mA at 2 bars and reaching only about 0.55 mA at 10 bars. The growth trend for aluminum is more gradual, with a noticeable acceleration beyond 5 bars. Overall, the results suggest that steel demonstrates higher responsiveness under increasing pressure compared to aluminum. A similar trend was observed for the measured voltage, which also increased with increasing inlet pressure as illustrated in Fig. 11(b). However, a clear difference is observed between the two materials.

Overall, the experimental results indicate that both turbine configurations respond positively to increasing inlet pressure, with corresponding increases in rotational speed and electrical output parameters. However, under the tested operating conditions, the turbine equipped with steel discs consistently produced higher rotational speeds and greater electrical power output than the aluminum configuration. The observed differences in performance between steel and aluminum discs cannot be attributed to a single parameter. Instead, they are likely influenced by a combination of factors, including material properties, disc spacing consistency, rotational inertia, the mechanical behavior of the rotating discs, and fluid interaction within the boundary layer of the airflow within the turbine. Further investigation, including mechanical, structural and flow analysis, is required to fully clarify and understand the underlying mechanisms and these effects.

Moreover, the electrical power output generated by the Tesla turbine was estimated based on the measured voltage and current according to each test condition. The electrical power output was calculated using the following relation:

9 $$\:P=V\times\:I$$

where *P* represents the measured electrical power output, *V* is the measured voltage, and *I* is the measured current.

**Fig. 12:**
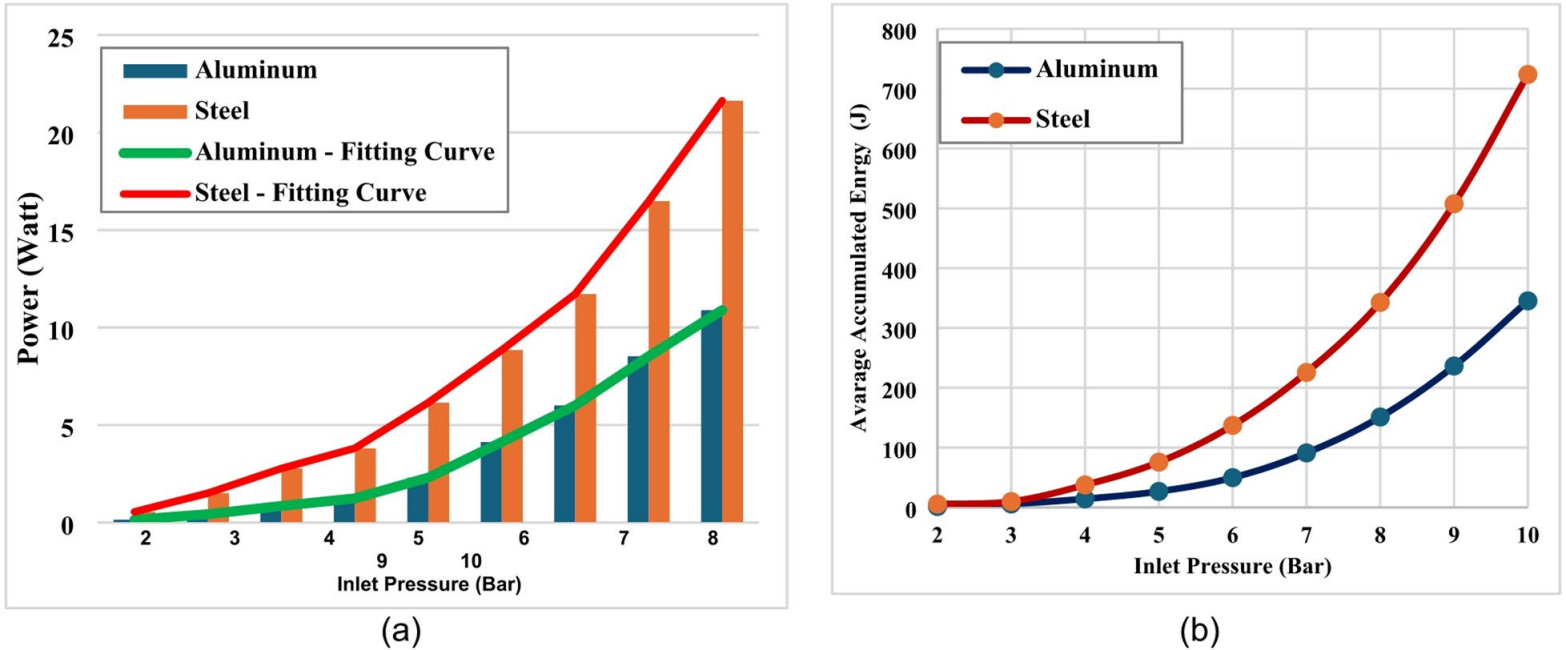
Average bar chart during the day measurements, (**a**) Power, (**b**) Accumulated energy.

The variation of electrical power output with respect to inlet pressure for two materials, aluminum and steel, in the pressure range of 2–10 bars are presented in bar charts as illustrated in Fig. 12(a). In both cases, power increases non-linearly with increasing inlet pressure, as represented by the fitting curves. At lower pressures (2–4 bar), both materials exhibit relatively small power values. However, as the inlet pressure rises, steel demonstrates a much sharper increase in power compared to aluminum. For instance, at 10 bars, the power output of steel reaches approximately 22 W, while aluminum achieves around 11 W, indicating that steel delivers nearly double the power of aluminum under the same conditions. The fitting curves confirm the exponential-like growth trend, with steel consistently positioned above aluminum throughout the pressure range. This behavior highlights the higher performance of steel in terms of electrical power output when subjected to higher inlet pressures.

In addition to the instantaneous electrical parameters, the electrical energy generated by the Tesla turbine was estimated based on the measured electrical power over a fixed operating period of 10 s for each test condition. The electrical energy was calculated using the relation:

10 $$\:E=P\times\:t$$

where *E* represents the electrical energy, *P* is the measured electrical power output, and *t* is the operating time.

Figure 12(b) illustrates the variation of the accumulated electrical energy generation with inlet pressure for both disc materials. In general, the results indicate that the generated electrical energy increases with increasing inlet pressure. This trend is consistent with the observed increase in rotational speed and electrical power output at higher pressure levels. For the turbine configuration equipped with steel discs, the generated electrical energy increased progressively as the inlet pressure increased from 2 bar to 10 bar. Under the same operating conditions, the turbine equipped with aluminum discs produced lower electrical energy values across the tested pressure range.

The difference in generated energy between the two configurations follows the same trend observed for rotational speed and electrical power output. Under the tested conditions, the turbine with steel discs consistently produced greater electrical energy compared with the aluminum configuration. These results further confirm that increasing inlet pressure enhances the electrical output of the Tesla turbine and that the choice of disc material influences the overall electrical performance of the system. However, additional investigations would be required to quantify the overall energy conversion efficiency of the system and to better understand the role of material properties in turbine operation.

Generally, the observed increase in rotational speed and electrical output with increasing inlet pressure can be explained by fundamental fluid dynamic and rotor interaction mechanisms. As the inlet pressure increases, the velocity of the incoming airflow also increases, resulting in a higher momentum flux entering the turbine. This enhances the viscous shear interaction between the fluid and the disc surfaces within the boundary layer, which is the primary operating principle of the Tesla turbine. Consequently, greater momentum is transferred to the rotor, leading to higher rotational speeds. In addition, the increased airflow velocity improves the effectiveness of tangential flow within the disc spacing, promoting more efficient momentum exchange. The combined effect of fluid acceleration, viscous drag, and rotor inertia contributes to the observed increase in electrical power output with pressure. These trends are consistent with the fundamental operating principles of boundary layer turbines.

Finally, the observed increase in rotational speed and power output with increasing inlet pressure is consistent with previously reported experimental studies on Tesla turbines, where higher pressure gradients enhance momentum transfer through boundary layer interaction. Similar trends have been reported in the literature, confirming that inlet pressure is a dominant parameter influencing turbine performance. This behavior can be attributed to the increase in tangential velocity of the airflow as the inlet pressure rises, which enhances viscous shear forces acting on the disc surfaces. As a result, greater momentum is transferred to the rotor, leading to higher rotational speeds and increased electrical power output.

Although the maximum electrical power output (~ 22 W at 10 bar) may appear relatively modest, it is important to note that the present study is based on a laboratory-scale prototype designed to demonstrate feasibility rather than maximize power generation. In practical applications, the total recoverable energy can be significantly increased by scaling the system or implementing multiple turbines in parallel within air brake systems. Additionally, such turbines can be installed at multiple discharge points, allowing cumulative energy recovery in large-scale transportation systems. Therefore, the proposed system has potential for practical implementation in decentralized energy recovery applications, particularly in heavy-duty vehicles and railway systems where compressed air is frequently discharged.

## Conclusion

Machines that convert fluid energy into rotational kinetic energy are classified as turbomachines. Among these systems, the Tesla turbine represents an unconventional bladeless turbomachine that operates based on the boundary layer adhesion principle. Owing to its structural simplicity and ease of manufacturing, the Tesla turbine has attracted attention as a potential device for small-scale and decentralized energy recovery applications.

In this study, a Tesla turbine prototype was designed and manufactured using CNC machining and experimentally evaluated for recovering waste compressed air energy from air brake systems used in heavy-duty vehicles and railway trains. The turbine consisted of ten coaxially arranged discs and was tested using two different disc materials, aluminum and steel, in order to examine the influence of material properties on turbine performance characteristics.

Experimental tests were conducted under inlet pressures ranging from 2 to 10 bar under both no-load and electrical load conditions. The rotational speed of the turbine and the generated electrical parameters, including voltage, current, and electrical power output, were measured and analyzed. The results show that increasing the inlet pressure leads to a noticeable increase in turbine rotational speed and electrical power output for both disc materials.

A comparison between the two configurations indicates that the turbine equipped with steel discs consistently produced higher rotational speeds and greater electrical power output than the aluminum configuration under the tested operating conditions. At lower inlet pressures, the turbine with aluminum discs was unable to generate measurable electrical output, whereas the turbine with steel discs continued to operate and produce electrical power. These results suggest that the mechanical properties of the disc material can influence the operational response of the turbine.

Overall, the experimental findings demonstrate the feasibility of employing a cost-effective Tesla turbine for recovering otherwise wasted compressed air energy in air brake systems. Such systems may contribute to improved energy utilization in transportation applications, particularly in scenarios where low-pressure energy sources are available. Future work will focus on optimizing turbine geometry, improving flow control conditions, and performing additional mechanical and structural analyses to better understand the influence of material properties on turbine behavior.

## Supplementary Information

Below is the link to the electronic supplementary material.

Supplementary Material 1

Supplementary Material 2

## Data Availability

The data that supports the findings of this study are provided within the manuscript file.
