# Supplementary material for: Experimental evaluation of a cost-effective tesla turbine for waste air energy recovery in transportation systems
Source: Sci Rep. 2026 May 14;16:15177. doi: 10.1038/s41598-026-48846-z (PMC13176347; doi:10.1038/s41598-026-48846-z)
Supplement: Supplementary file 1 — Supplementary Material 1 [file 41598_2026_48846_MOESM1_ESM.pdf]

Highlights

- A cost-effective Tesla turbine was developed for waste compressed air energy recovery.
- The turbine was experimentally evaluated under inlet pressures ranging from 2 to 10 bars.
- Disc material significantly affects rotational speed and electrical power output.
- Steel discs achieved higher efficiency and stable power generation compared to aluminum.
- The system demonstrates strong potential for decentralized energy recovery in air brake systems.
