# Supplementary figures and images for: Experimental evaluation of a cost-effective tesla turbine for waste air energy recovery in transportation systems
Source: Sci Rep. 2026 May 14;16:15177. doi: 10.1038/s41598-026-48846-z (PMC13176347; doi:10.1038/s41598-026-48846-z)

# Graphical Abstract

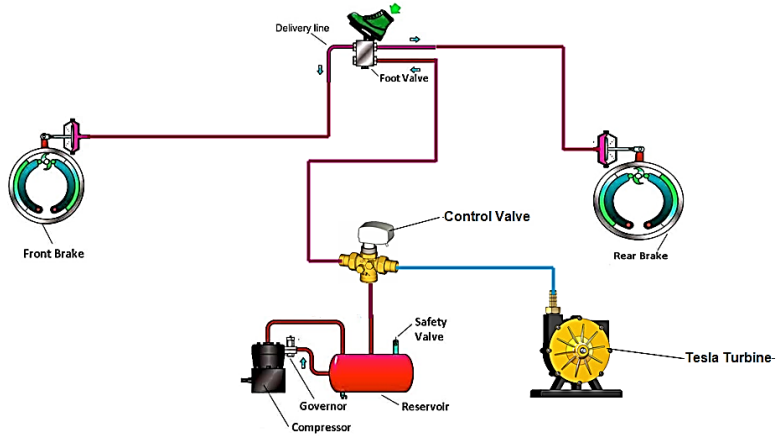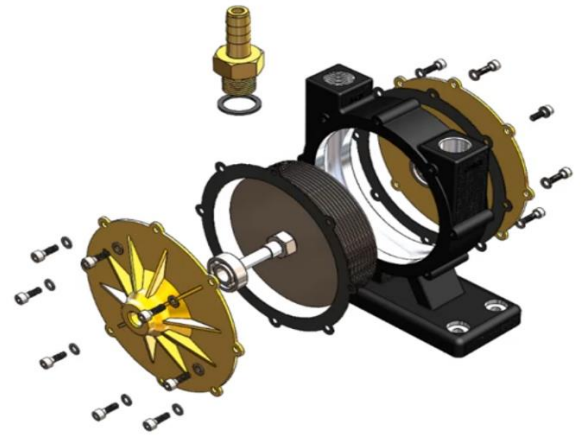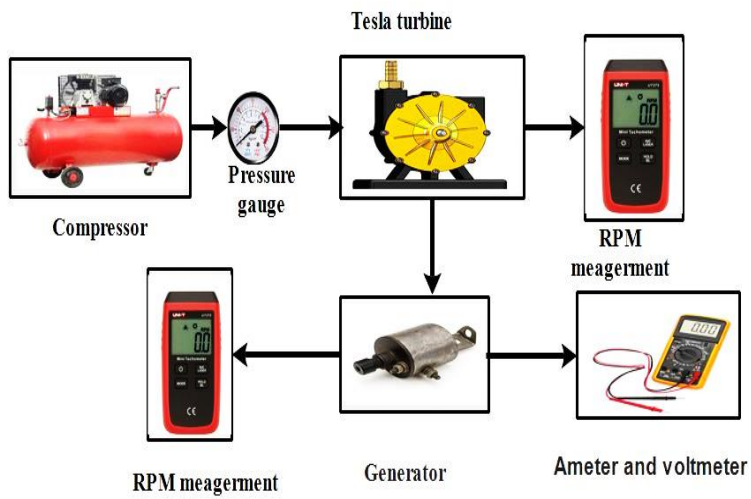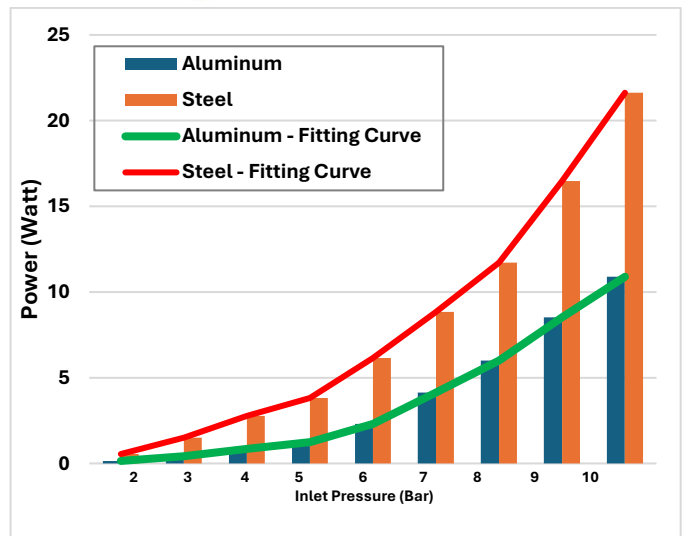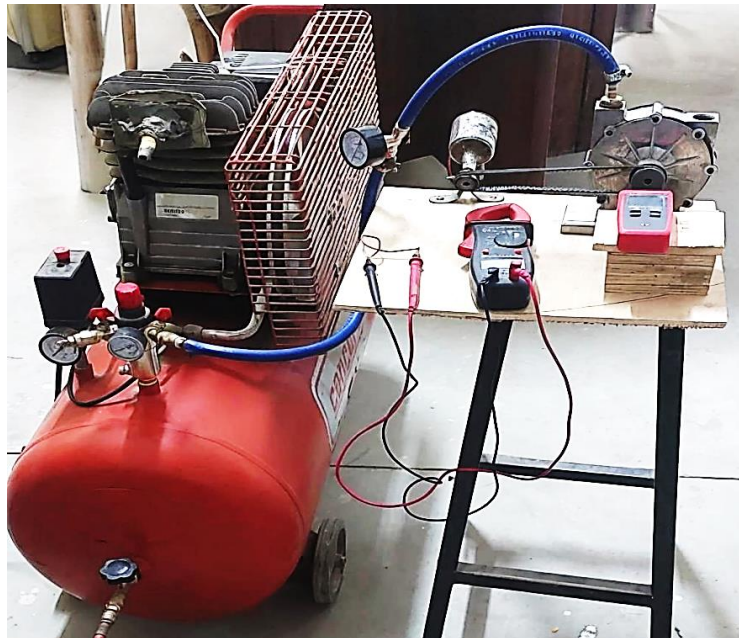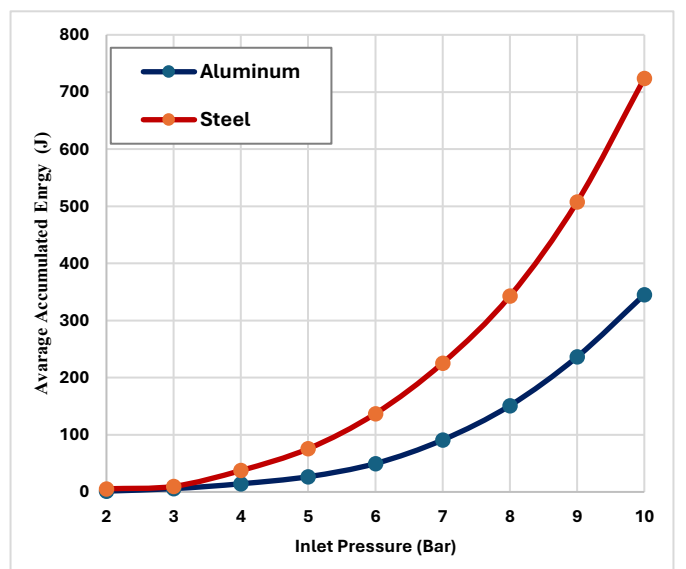

Supplement: Supplementary file 2 — Supplementary Material 2 [file 41598_2026_48846_MOESM2_ESM.pdf]
